# Polyparasitism Is Associated with Increased Disease Severity in *Toxoplasma gondii*-Infected Marine Sentinel Species

**DOI:** 10.1371/journal.pntd.0001142

**Published:** 2011-05-24

**Authors:** Amanda K. Gibson, Stephen Raverty, Dyanna M. Lambourn, Jessica Huggins, Spencer L. Magargal, Michael E. Grigg

**Affiliations:** 1 Laboratory of Parasitic Diseases, National Institutes of Health, National Institute of Allergy and Infectious Diseases (NIAID), Bethesda, Maryland, United States of America; 2 Animal Health Centre, Ministry of Agriculture and Food, Abbotsford, British Columbia, Canada; 3 Marine Mammal Research Unit, Fisheries Centre, Aquatic Ecosystems Research Laboratory (AERL), University of British Columbia, Vancouver, British Columbia, Canada; 4 Washington Department of Fish and Wildlife, Lakewood, Washington, United States of America; 5 Cascadia Research Collective, Olympia, Washington, United States of America; University of California San Diego School of Medicine, United States of America

## Abstract

In 1995, one of the largest outbreaks of human toxoplasmosis occurred in the Pacific Northwest region of North America. Genetic typing identified a novel *Toxoplasma gondii* strain linked to the outbreak, in which a wide spectrum of human disease was observed. For this globally-distributed, water-borne zoonosis, strain type is one variable influencing disease, but the inability of strain type to consistently explain variations in disease severity suggests that parasite genotype alone does not determine the outcome of infection. We investigated polyparasitism (infection with multiple parasite species) as a modulator of disease severity by examining the association of concomitant infection of *T. gondii* and the related parasite *Sarcocystis neurona* with protozoal disease in wild marine mammals from the Pacific Northwest. These hosts ostensibly serve as sentinels for the detection of terrestrial parasites implicated in water-borne epidemics of humans and wildlife in this endemic region. Marine mammals (151 stranded and 10 healthy individuals) sampled over 6 years were assessed for protozoal infection using multi-locus PCR-DNA sequencing directly from host tissues. Genetic analyses uncovered a high prevalence and diversity of protozoa, with 147/161 (91%) of our sampled population infected. From 2004 to 2009, the relative frequency of *S. neurona* infections increased dramatically, surpassing that of *T. gondii*. The majority of *T. gondii* infections were by genotypes bearing Type I lineage alleles, though strain genotype was not associated with disease severity. Significantly, polyparasitism with *S. neurona* and *T. gondii* was common (42%) and was associated with higher mortality and more severe protozoal encephalitis. Our finding of widespread polyparasitism among marine mammals indicates pervasive contamination of waterways by zoonotic agents. Furthermore, the significant association of concomitant infection with mortality and protozoal encephalitis identifies polyparasitism as an important factor contributing to disease severity in marine mammals.

## Author Summary

Severity of toxoplasmosis, a water-borne zoonosis, varies widely from chronic and benign to acutely fatal. Here, we investigate polyparasitism (infection with multiple parasite species) as one factor governing the spectrum of disease in *Toxoplasma gondii* infections. This study utilized wild marine mammals as sentinels to detect contamination of waterways by *T. gondii* and a similar protozoan, *Sarcocystis neurona*, which have been linked to water-borne outbreaks in humans and wildlife along North America's Pacific Coast. Using genetic tools, we found high rates of protozoal infection, predominantly concomitant infections, in animals inhabiting major waterways of the Pacific Northwest. These dual infections of *T. gondii* and *S. neurona* were more frequently associated with mortality and protozoal encephalitis than single infections, indicating a role for polyparasitism in disease severity. Finally, rare *T. gondii* genotypes linked to a major human outbreak in the Pacific Northwest were abundant in marine mammals of the region, emphasizing wildlife as relevant sentinels for evaluation of human health risks. Our data implicate polyparasitism as a critical factor associated with the severity of protozoal disease. We also identify the need for vigilant surveillance of public waterways to prevent fecal contamination recurrently threatening human and wildlife health along the Pacific coast.

## Introduction

A single individual often plays host to not only one, but to an entire community of parasites [1]–[3], and this polyparasitism has been identified as a critical factor in determining the virulence of an infection [4]–[6]. The study of host-parasite interactions has accordingly come to question the one-host, one-parasite paradigm and to emphasize a multi-host, multi-parasite approach to infectious disease research and control.

For *Toxoplasma gondii,* a tissue-encysting coccidian parasite within the phylum Apicomplexa, infections are typically chronic and benign but can cause acute disease and mortality. Previous research has primarily focused on parasite strain as a factor influencing the severity of toxoplasmosis. The majority of associations between virulence and strain type are founded upon infections of mouse models by three clonal lineages, referred to as Types I, II, and III, that are most commonly associated with human infections in Europe and North America [7], [8]. Type II and III infections in mice are classified as avirulent and Type I as acutely virulent [9], [10]. However, studies have increasingly questioned this strict dichotomy [11]; mouse virulent Type II strains and avirulent Type I-like strains have been discovered [12], [13], while acute human toxoplasmosis is commonly attributed to Type II and atypical strains [7], [8], [11], [14]–[16]. Similarly, acute disease in alternate hosts is associated with different strain types. Significant mortality of Southern sea otters (*Enhydra lutris nereis*) between 1998 and 2004 was exclusively associated with Type II and Type X, a novel clade of strains first identified in sea otters [17].

This variability in disease within a given strain type indicates that genotype alone does not dictate the severity of toxoplasmosis. Research in other systems, most notably in human populations of non-industrialized nations, have found polyparasitism to be the rule rather than the exception, and the interaction of multiple pathogens can significantly impact the course and severity of an infection [18]. For example, most neglected tropical diseases (NTDs) overlap geographically with the big three (HIV/AIDS, malaria, and tuberculosis), for which concomitant infection can have profound implications for susceptibility, transmission, mortality, and vaccine efficacy [3], [19]–[21]. Toxoplasmic encephalitis is a common and often fatal complication in HIV/AIDS, occurring in as many as 50% of HIV/AIDS patients in some areas [22]. Accordingly, polyparasitism may be a critical variable that has yet to be assessed in the study of *T. gondii* epidemiology and disease, since laboratory studies of concomitant infection with *T. gondii* have rarely been extended to the clinic or the field.

Here, we assess the frequency of polyparasitism with the protozoal parasites *T. gondii* and *S. neurona* infecting wild marine mammals of the Pacific Northwest to specifically investigate whether concomitant infection influences protozoal disease severity. These parasites have joined the ranks of bacterial (e.g. *Brucella* [23]), viral (e.g. herpesvirus [24]), and fungal (e.g. *Cryptococcus gattii* [25]) zoonotic agents that are classified as pollutogens, terrestrial pathogens increasingly found to cause disease in marine organisms [26]. For marine mammals, *T. gondii* has been associated with population suppression [27], [28], and in 2004, an epizootic of *S. neurona* caused mortality of ∼1.5% of the Southern sea otter population in California [29], [30]. North America's Pacific Northwest represents a particularly relevant niche for the study of protozoal disease. *Toxoplasma gondii* emerged as an important water-borne zoonosis in this region in 1995, when contamination of a Vancouver Island drinking-water reservoir with *T. gondii* oocysts resulted in at least 2894 human infections, 100 of which were acutely symptomatic [31]. As pollution and urban development erode land-to-sea barriers, marine mammals have come to serve as sentinels for the detection of contaminants polluting our shared environment (e.g. waterborne zoonoses) and can act as valuable surrogates for the study of emerging disease processes [27], [32].

This study of a natural system combines epidemiological approaches with genetic tools and histopathology to survey a region endemic for protozoal parasites and thus discover factors governing the emergence of disease. We determined the contribution of concomitant infection to symptomatic disease by genetically characterizing a population of tissue-encysting coccidia infecting marine mammals. Marine mammals (151 stranded and 10 healthy individuals) were collected along the coasts of Oregon, Washington, and southern British Columbia over a 6-year period, from 2004 to 2009. Pathology reports, geographic and temporal data, and direct molecular detection of coccidian parasite DNA in tissues collected from a range of marine mammal species were combined to identify risk factors for disease. Our findings provide new insight into the role of polyparasitism in modulating the severity of protozoal disease and support the study of disease in wildlife, notably marine mammals, as indicators of human and ecosystem health.

## Methods

### Ethics statement

Animal carcasses were gathered and samples processed as part of the Northwest Marine Mammal Stranding Network activities authorized under Marine Mammal Protection Act (MMPA) Stranding Agreements (SA) and Section 109(h) (16 U.S.C. 1379(h)). Additional specimens were acquired under a National Marine Fisheries Service (NMSF) MMPA Section 120 Letter of Authorization and NMFS MMPA Scientific Research Permit 782–1702. Throughout the course of this study, appropriate United States CITES export and Canadian CITES import permits were obtained, and United States Fish and Wildlife declarations were completed for cross border transport of case material. No live animals were used in the diagnostic investigation of this case series.

### Sample collection

Between 2004 and 2009, over 6000 marine mammals stranded on beaches along the coastal areas of Oregon, Washington, and southern British Columbia, Canada and along the inland waters of Washington and southern British Columbia. Of these strandings, tissues were available from 151 individuals that had been identified as suspect protozoal encephalitis cases based upon observed ante-mortem neurologic signs (e.g. depression, lack of response to human approach, opisthotonous, seizure, paralysis and ataxia) and upon post-mortem condition for those animals found dead. In addition, stranded Guadalupe fur seals (*Arctocephalus townsendi*) and harbor porpoises (*Phocoena phocoena*) were submitted as suspect protozoal cases due to region-wide unusual mortality events. Finally, sea otters (*Enhydra lutris*) were submitted as suspect protozoal cases due to previous outbreaks of protozoal disease in California sea otter populations.

Tissues, including heart, brain, muscle and lymph nodes, from the 151 suspect protozoal cases were submitted for genetic screening along with 10 healthy adult California sea lion (*Zalophus californianus*) males that had traveled 145 miles up the Columbia River from the outer coast to Bonneville Dam, Oregon-Washington and were euthanized to protect fish stocks. Hence, this study investigated a total of 161 individuals, representing a non-random sample of stranded, suspect protozoal cases and a small, non-random population of healthy individuals. Specifically, animals were sampled from the inland waters of Washington and southern British Columbia, Canada, encompassing the regions of the south Puget Sound, WA (n = 62), Strait of Juan de Fuca (n = 6), and San Juan Islands/Eastern Bay (n = 7), as well as from the outer coasts of southern British Columbia, Washington and Oregon (n = 75) and from the Columbia River, Bonneville Dam (n = 10). One migratory Killer whale (*Orcinus orca*) from Northern California, outside of the range specified for all other animals, was also sampled.

The marine species tested included 12 California sea lions, 1 northern elephant seal (*Mirounga angustirostris*), 13 Guadalupe fur seals, 81 harbor seals (*Phoca vitulina*), 7 Steller sea lions (*Eumetopias jubatus*), 36 harbor porpoises, 1 harbor/Dall's porpoise hybrid (*Phocoena phocoena x dalli*), 1 Killer whale, 1 Pacific white-sided dolphin (*Lagenorhynchus obliquidens*), 2 Pygmy sperm whales (*Kogia breviceps*), 1 sperm whale (*Physeter macrocephalus*), and 5 Northern sea otters. Individuals ranged in age from adults to fetuses. Five fetuses, in addition to their mothers, were included in the data set.

### Genetic analysis

Tissues were stored at −20°C prior to receipt and time of use. DNA extractions were conducted using the spin-column protocol for purification of total DNA from animal tissues (Qiagen DNeasy Blood & Tissue kit). DNA was eluted in 30 µl of a 1∶10 dilution of Qiagen EB buffer. Extracted DNA samples were stored at −20°C between PCR reactions.

Sequences for primers used in PCR amplification are reported in Table 1. Pan-coccidian primers, anchored in the 18S and 5.8S small subunit (SSU) rDNA gene array, that amplify across the internal transcribed spacer 1 (ITS1) region were used to distinguish among closely related and novel species of tissue-encysting coccidian parasites (Miller, R.H. and Grigg, M.E., personal communication). The *T. gondii* genome contains 110 identical gene copies within the rDNA locus [33]. ITS1 copy numbers for the other tissue-encysting coccidia reported are currently unknown. The presence of *S. neurona* DNA was detected with the ITS1_500_ primers, which specifically amplify a ∼500 base-pair portion of the *S. neurona* or *S. falcatula* ITS1 region [29].

**Table 1:** Sequences of nested primer sets used in this study.

Marker		External 5′ to 3′	Internal 5′ to 3′
**ITS1** a	*Forward*	TTA CGT CCC TGC CCT TTG TA	GTG AAC CTT AAC ACT TAG AGG
**ITS1** a	*Reverse*	TGC GTT CTT CAT CGT TGC GC	GAG CCA AGA CAT CCA TTG CT
**ITS1_500_** b	*Forward*	TTCTCTTGTGTGTGCCCCTAC	CAAAATGAACGTGTCTATGTGTGA
**ITS1_500_** b	*Reverse*	TGCGTCC TTCATCGTTGCGC	GAGCCAAGACATC CATTGCT
***B1*** c	*Forward*	TGT TCT GTC CTA TCG CAA CG	TCT TCC CAG ACG TGG ATT TC
***B1*** c	*Reverse*	ACG GAT GCA GTT CCT TTC TG	CTC GAC AAT ACG CTG CTT GA
***NTS2*** d	*Forward*	TGT GCT CGT GAC TTG ATG TG	ACC AGA AAG CCA GTG GAA TG
***NTS2*** d	*Reverse*	GGA AAA CAA GCG GTG AGA TT	TTT GTT TTT CTC GCG TAG AGG
**5′** ***SAG1*** e	*Forward*	GTT CTA ACC ACG CAC CCT GAG	CAA TGT GCA CCT GTA GGA AGC
**5′** ***SAG1*** e	*Reverse*	GTG GTT CTC CGT CGG TGT GAG	TTA TCT GGG CAG GTG ACA AC

a Miller, R.H. and Grigg, M.E. (personal communication)

b Miller et al. (2009)

c Grigg and Boothroyd (2001)

d Bottós et al. (2009)

e Burg et al. (1988)

Genotyping of *T. gondii* was accomplished using two multi-copy screening loci, *B1* and *NTS2*, for which primer sets have been described previously by Grigg and Boothroyd [34] and Bottós [35], respectively. The *B1* locus, a tandemly-arrayed 35-fold repetitive gene, is considered a sensitive and specific region for distinguishing among archetypal strain alleles [34], [35]. Similarly, *NTS2* (Non Transcribed Spacer 2) is a polymorphic region of the rDNA gene array that can be used to discriminate between archetypal *Toxoplasma* alleles [35]. Genotyping at the single-copy *SAG1* locus, described by Burg [36], was performed on those samples positive at both *B1* and *NTS2* loci. Further genetic screening was not conducted due to the difficulty in amplifying parasite DNA from environmental samples, as opposed to parasite isolates. All PCR reactions were nested, and all PCR products were sent for DNA sequencing.

PCR amplification, product visualization, and DNA sequencing were conducted according to Wendte [30]. Amplifications of the *B1* and *NTS2* regions were modified to consist of 35 cycles of 94°C for 5 min, 94°C for 40 s, 58°C for 40 s, 72°C for 40 s and 72°C for 10 min. Sequencing was performed by Rocky Mountain Laboratory Genomics Unit DNA Sequencing Center, Division of Intramural Research, Hamilton, Montana.

The Seqman component of the Lasergene software was used to align and analyze sequences. The identity of sequences was verified via alignment to known reference sequences and a nucleotide BLAST search in GenBank.

### Geographic distribution

Location data, as both descriptive locations and geographic coordinates, were obtained for all sampled animals. The distribution map was generated in ArcGIS Desktop 10. Geographic coordinates were adjusted to visually clarify the number and distribution of sampled animals. Sampled individuals were grouped into the outer coast (outer coast of southern British Columbia, Washington, and Oregon, including the Columbia River) or inland waters of Washington and southern British Columbia (south Puget Sound, Strait of Juan de Fuca, and San Juan Islands/Eastern Bay) populations. These groupings are supported by previous studies investigating the population structure of regional harbor seals and further suggested by similar work in harbor porpoises [37], [38]. Rates of infection between the inland waters and the outer coast were compared using a Chi-square analysis. All statistical analyses were conducted with the software R v2.11.0.

### Phylogenetic reconstruction

The ITS1 region of the small subunit ribosomal gene array, rather than the 18S region, was used in phylogenetic reconstruction due to the inability of the 18S region to resolve closely-related species of tissue-encysting coccidia. Reference sequences for the ITS1 region of known coccidian parasites were obtained from GenBank. ITS1 sequences for novel coccidia were obtained by sequencing PCR products amplified from tissues of infected animals. Accession numbers for all sequences are reported in Table S1.

SATé-II was used to align sequences, as described in Liu [39] (http://phylo.bio.ku.edu/software/sate/sate.html). Sub-alignments were constructed with MAFFT and merged with OPAL. The maximum subproblem fraction was set to 5, and a centroid decomposition approach was applied. The SATé-II alignment file was then used to construct a phylogeny in MEGA4. The Neighbor-Joining method was applied, with evolutionary distances computed using a Maximum Composite Likelihood approach and pairwise deletion of gaps and missing data. A bootstrap test of 500 replicates was conducted in MEGA4. Editing was performed in FigTree v1.3.1 and Inkscape v0.48.

### Temporal dynamics

To assess the variability in time of rates of infection with protozoal parasite species, relative rates of total *S. neurona* infections versus total *T. gondii* infections were compared between early and late years of collection. “Early” was defined as the 4-year collection period between 2004 and 2007 while “late” was defined as 2008–2009. Collection years were partitioned as such based upon evident changes in parasite population structure in 2008. The same temporal variability in infection was analyzed for resident populations of the outer coast and inland waters in order to investigate the contribution of local environmental contamination to infection patterns. Harbor seals and harbor porpoises were defined as resident individuals, while individuals of all other species, which are largely migratory, were considered non-residents and excluded.

To test whether the relative frequencies of protozoal species varied with the relative proportions of host taxa collected, individuals were classified as belonging to one of two host taxa, suborder Pinnipedia (e.g. seals, sea lions) or order Cetacea (e.g. whales, dolphins, porpoises). Relative rates of infection of the two taxa were then compared, as were the relative proportions of taxa collected in 2004–2007 and 2008–2009. Northern sea otters (family Mustelidae) were excluded from these analyses due to low representation of their taxonomic group. Chi-square analyses were used for all comparisons, and statistical analyses were conducted as outlined above.

### Histopathology grading

A gross necropsy was performed on all collected animals in suitable post-mortem condition. Representative samples from all major tissues were fixed in 10% neutral buffered formalin for processing and histopathologic evaluation. Tissues were paraffin embedded, and 5 µm sections were cut and stained with haematoxilin and eosin (H&E). For H&E-stained sections of tissues in which coccidian parasites were identified by histopathology, immunohistochemistry was performed to verify the presence of *Toxoplasma gondii* (rabbit polyclonal, AR125-5R; Biogenex Laboratories, Inc., San Ramon, California) and *Sarcocystis neurona* (rabbit polyclonal, G. Barr, University of California, Davis, California).

Histopathology grading was focused primarily on brain tissue to assess the degree of protozoal encephalitis present in all animals that exhibited ante-mortem neurological signs or were otherwise classified as suspect protozoal cases [40]. Of the individuals testing positive for *S. neurona* and/or *T. gondii* infection, brain sections from 108 were suitable for microscopic and histopathology investigation. To ascertain the contribution of protozoal infection to neuropathology and morbidity, histopathology grading was conducted using two criteria: 1) the presence and number of protozoa and 2) the extent and severity of associated inflammatory infiltrate in the brain. By combining parasite load with degree of inflammation and other ancillary diagnostic laboratory results, protozoal infection was classified as an immediate (primary), contributing, or incidental (auxiliary) cause of encephalitis and death. In a subset of 83 PCR-positive individuals, the brain was in sufficient condition to score the degree of protozoal encephalitis, ranked as 0 (absent), 1 (mild), 2 (moderate), 3 (marked), and 4 (severe). Histopathology and protozoal encephalitis assessments were limited to a subset of the infected hosts due to variations in the state of decay, physical trauma, intercurrent disease, and the interval between death and tissue collection. Accordingly, a small number of individuals, comprising in utero fetuses and aborted fetuses, were excluded from this analysis due to difficulties in determination of cause of death and pathology. Pups that had completed the gestational period were, however, retained in the analysis. Finally, analysis of disease severity was not performed for animals infected with coccidian parasites other than *T. gondii* and *S. neurona* due to insufficient sampling.

In analyzing protozoal infection as a cause of death, cases with protozoal infection as an immediate cause of death were contrasted with those for which protozoal infection was a contributing or an incidental cause of death. In analyzing protozoal encephalitis, the 5 levels of severity were collapsed into two categories, with 0–2 indicating absent to moderate encephalitis and 3–4 representing more severe encephalitis. Severity of single vs. dual protozoal infections, as measured by rates of immediate cause of death and of marked/severe encephalitis, was compared with Chi-square analyses, as outlined above.

Severity of protozoal disease was also compared between resident individuals of the outer coast versus inland waters (as previously defined). Rates of protozoal infection as an immediate versus a contributing or incidental cause of death were compared between the two populations using a Chi-square analysis. The same was done for rates of severe/marked (4–3) versus moderate/mild/absent (2–0) protozoal encephalitis.

### Toxoplasma gondii molecular characterization

Animal tissues identified as infected with *T. gondii* were screened using primers against the *B1*, *NTS2*, and *SAG1* loci. Genotype was determined by comparison to reference sequences derived from archetypal Types I, II, III, and X strains. Alleles were classified either as Type I, II/III, or X or as Type I-like, II/III-like or X-like, represented by U_I,_ U_II/III,_ or U_X_, respectively, when polymorphisms in addition to the Type-defining polymorphisms were present. Sequences that were sufficiently divergent from Type I, II/III, and X alleles were designated as unique alleles (U). Sequences are henceforth referred to as genotypes, and genotype is defined based upon allelic identity at one to three of the loci investigated here.

Network diagrams of the *B1* and *NTS2* loci were constructed using the Templeton [41] network estimation procedure for visualization of the *T. gondii* population structure. Implemented in the TCS v.1.21 software of Clement [42], this approach serves to estimate relationships among alleles within a population when allelic diversity is low, ancestral alleles are extant, and recombination is possible. The TCS software generates a parsimonious network of relationships between alleles in the population and calculates the frequency of each allele. The output files were visually modified for presentation and analyses. When two sequences were detected for the same individual (i.e. multiple infections), both sequences were chosen to represent that individual in the TCS analyses. For these reasons, the population of alleles presented in the network diagrams differs slightly from the total population of alleles. Network diagrams for the *SAG1* locus were not constructed due to low sample size.

Comparisons of symptomatic disease were made with Fisher's Exact test, by comparing mortality and protozoal encephalitis between concatenated *T. gondii* genotypes (classified as Types I, II, X, and multiple infections) (Table S2). Atypical genotypes were excluded due to uncertainty over infection type (unique genotype or multiple infection). Concatenated genotypes at multiple loci, rather than alleles at single loci, were used to allow comparison of disease severity of multiple infections.

Host specificity of *T. gondii* genotype was investigated by comparing, with Fisher' Exact test, the frequency of genotype infection across two host taxa, suborder Pinnipedia and order Cetacea. For this analysis, genotype was defined by individual alleles at the *B1* and *NTS2* loci, for which independent analyses were conducted. Groupings of alleles were based upon the relationships generated by the TCS network estimation procedure. Individual alleles at single loci, rather than concatenated genotypes across multiple loci, were used in order to provide a larger, more accurate sampling and to account for the identity of all genotypes infecting a single individual in the case of a multiple infection.

## Results

### Prevalence and distribution of protozoal parasitism

**Figure 1:**
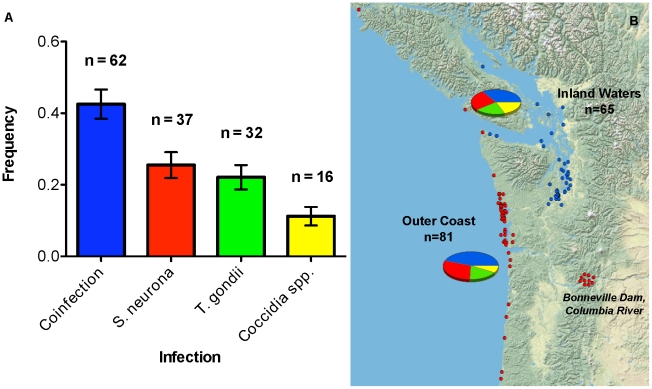
Polyparasitism dominates infection in marine mammals of the Pacific Northwest. (A) Frequency of parasite infection type based upon molecular genotyping analyses performed on 147 stranded marine mammals infected with tissue-encysting coccidia. Dual infections of *S. neurona* and *T. gondii* (blue), single infections of *S. neurona* (red) and of *T. gondii* (green), and infections with previously undescribed coccidian species (yellow) were detected. Sample sizes for each infection type are given, and error bars indicate the standard error of the proportion. Graph generated in Prism v5.0a. (B) Distribution of infected marine mammals across Washington, Oregon, and southern British Columbia, Canada. Each circle represents a single sampled individual; outer coast individuals are marked in red and inland waters individuals in blue. The proportions of dual infections, *S. neurona* single infections, *T. gondii* single infections, and unique coccidian infections are shown for both the outer coast and inland waters regions, with the size of each chart directly proportional to the number of infected individuals collected from the region. The 10 inland individuals represent healthy California sea lion males that traversed the Columbia River from the outer coast to the Bonneville Dam. Geographic coordinates were adjusted to visually clarify the number and distribution of sampled animals. A single sample from Northern California is excluded. Distribution maps and charts were constructed in ArcGIS Desktop 10.

**Figure 2:**
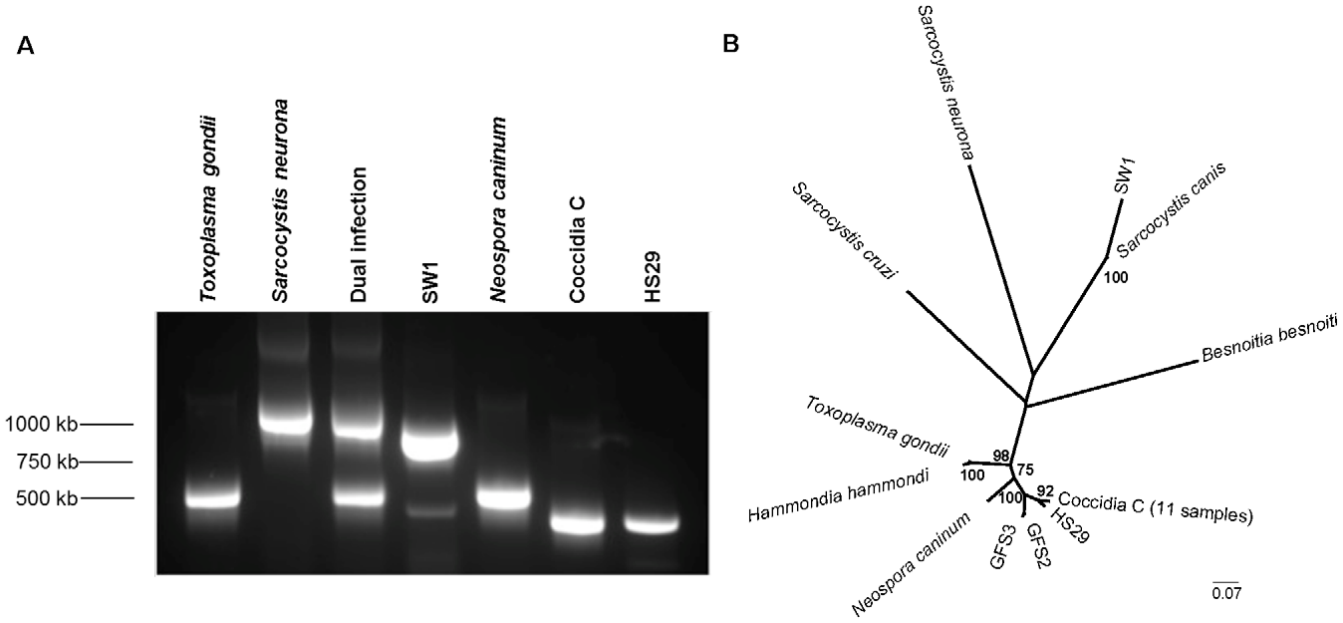
Direct amplification of the ITS1 region from infected tissues detects several unique tissue-encysting coccidia. (A) Species of tissue-encysting coccidia can be visually distinguished by size polymorphisms within the ITS1 region of the small subunit (SSU) rDNA nuclear gene array. Parasite DNA was amplified using nested primers designed to amplify across the ITS1 region of all tissue-encysting coccidian parasites. PCR products were separated via electrophoresis in a 1.5% agarose gel and visualized using GelRed. Shown here are the ITS1 amplicons of three known coccidian species (*T. gondii*, *S. neurona*, and *N. caninum*), a dual infection of *T. gondii* and *S. neurona*, and three previously undescribed coccidia (SW1, Coccidia C, and HS29). (B) Phylogenetic reconstruction based upon maximum likelihood distances of DNA sequences for the ITS1 region of known and novel tissue-encysting coccidian parasites (*S. canis-*like SW1, *N. caninum*-like Coccidia C, GFS2, GFS3, HS29). *Besnoitia besnoitii* was chosen as the outgroup. Sequences were obtained from GenBank NCBI or by direct sequencing of amplified parasite DNA. Accession numbers are reported in Table S1. The Neighbor-Joining method, with gaps treated as pairwise deletions, was used, and 500 replicate bootstrap calculations are shown for clades with scores over 70.

Four hundred and ninety-four tissues from 161 individuals were screened for protozoal infection; 147 individuals (91%) tested positive. Of the 10 adult California sea lion males sampled from the Columbia River near Bonneville Dam, representing healthy, euthanized individuals, all were infected with either *S. neurona* singly (n = 8), *T. gondii* singly (n = 1), or *T. gondii* and *S. neurona* dually (n = 1). Of the remaining 151 suspect protozoal cases, 121 were infected either with *S. neurona* (n = 29), *T. gondii* (n = 31), or both *T. gondii* and *S. neurona*, (n = 61). The combined distribution of protozoal infections was therefore 37 *S. neurona* infections, 32 *T. gondii* infections, and 62 *S. neurona* and *T. gondii* dual infections (Fig. 1a). In this set of 131 PCR-positive cases, 7 animals were also co-infected with another coccidian parasite (in addition to *T. gondii* and/or *S. neurona*). The remaining 16 PCR-positive protozoal cases (for a total of 147) were not infected with either *T. gondii* or *S. neurona,* but rather with a variety of known or novel tissue-encysting coccidian parasites (see below). Size polymorphism in the ITS1 region (Fig. 2a) was used to readily distinguish between single *T. gondii*, single *S. neurona*, and dual infections. All PCR products were verified by DNA sequencing.

Approximately equal numbers of individuals with protozoal infections were identified in the outer coast (n = 81) and the inland waters (n = 65). One individual, a dually infected Killer whale, was collected outside of these two regions, in Northern California. In Figure 1b, pie charts showing the proportions of dual infections (blue), *S. neurona* single infections (red), *T. gondii* single infections (green), and unique coccidian infections (yellow) can be seen for both regions, with chart size scaled by sample size per region. The rates of *T. gondii*, *S. neurona*, and dual infections were indistinguishable between the populations of the outer coast and the inland waters (χ2 = 1.2731, DF = 2, p = 0.5291).

For the 23 individuals found infected with other coccidian parasites (16 single and 7 *T. gondii* and/or *S. neurona* cases), agarose gel electrophoresis differentiated unique tissue-encysting coccidia from *T. gondii* and *S. neurona* by size polymorphism at the ITS1 locus (Fig. 2a: SW1, Coccidia C, HS29). DNA sequencing confirmed their identity. Five individuals were infected with the closely related parasite *Neospora caninum*. A novel DNA sequence with similarity to *S. canis* was amplified from the single sperm whale (Fig. 2b: SW1), and two other highly divergent sequences were amplified from tissues of a California sea lion and a harbor seal. Fifteen cases of protozoal infection were by a unique clade of coccidia possessing significant DNA sequence homology (max identity of 86–94% on BLASTn) to *N. caninum*. Eleven of these novel DNA sequences were identical and are referred to as “Coccidia C” in this study (Fig. 2b). Ten of the 11 Coccidia C DNA sequences were amplified from harbor seals of the south Puget Sound (inland waters), and all but one infected animal was collected between 2007 and 2009. Three of the 15 sequences were amplified from Guadalupe fur seals (GFS1, GFS2, GFS3) from the outer coast; these sequences were highly similar to Coccidia C at the ITS1 locus (Fig. 2b). Finally, a unique ITS1 sequence was amplified from a harbor seal; it shared DNA sequence homology with both *N. caninum* and Coccidia C (Fig. 2b: HS29). These sequences likely represent infections with new species of coccidian parasites infecting marine mammals and have been deposited in GenBank (see table S1 for accession numbers).

### Temporal variation in infection

**Figure 3:**
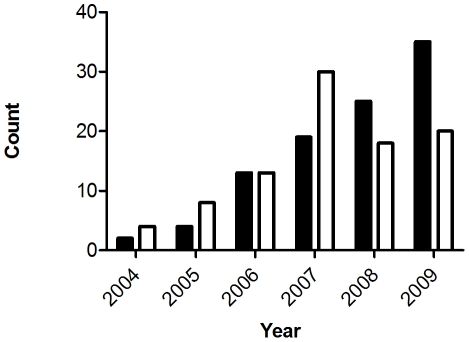
Frequencies of *Toxoplasma gondii* and *Sarcocystis neurona* shift dramatically over a short time period. The relative frequencies of *S. neurona* (black) and *T. gondii* (white) infections in the Pacific Northwest are shown for years 2004 to 2009. *Sarcocystis neurona* replaced *T. gondii* in 2008 as the more prevalent protozoal pathogen. Graph generated in Prism v5.0a.

When rates of total *T. gondii* infection (i.e. single *T. gondii* infections plus *T. gondii* and *S. neurona* dual infections) versus total *S. neurona* infection were compared over the 6 years of the study, the proportion of *S. neurona* infections increased steadily from 2004 to 2009. In contrast, *T. gondii* infections peaked in 2007 then declined relative to *S. neurona* (Fig. 3). In 2008, *S. neurona* replaced *T. gondii* as the major agent of protozoal infection, and the relative proportions of *S. neurona* to *T. gondii* infections in 2008–2009 differed significantly from those in 2004–2007 (χ2 = 7.1267, DF = 1, p = 0.008). The same increase in *S. neurona* and decrease in *T. gondii* infections from 2004 to 2009 was present when only resident individuals (harbor seals and harbor porpoises) were considered, and the difference in the relative proportions of *S. neurona* to *T. gondii* in 2004–2007 vs. 2008–2009 was marginally significant (χ2 = 3.4921, DF = 1, p = 0.06166).

We investigated the contributions of environmental load and host-specificity to this temporal shift in tissue-encysting coccidian species. Pinnipeds and cetaceans represent the majority of the sampled population and are highly divergent mammalian orders that could conceivably differ in their susceptibility to infection with *T. gondii* and *S. neurona*. Accordingly, the variation in the relative frequency of protozoal species identified could reflect temporal variation in the host taxa collected. Analyses, however, did not support this hypothesis. The relative rates of infection with *S. neurona* and *T. gondii* were indistinguishable between cetaceans and pinnipeds (χ2 = 9e-04, DF = 1, p = 0.976), with the two protozoal species each representing ∼50% of infections for both host groups. Furthermore, the relative proportions of pinnipeds and cetaceans collected over 2004–2007 and 2008–2009, the two time periods that define the parasite population shift shown here, were identical (χ2 = 0.0237, DF = 1, p = 0.876). These findings support changes in the environmental load of coccidian parasites, as opposed to host-specificity and shifts in animal collections, as the underlying mechanism for the temporal dynamism of protozoal parasites infecting marine mammals.

### Histopathology grading of protozoal disease

**Figure 4:**
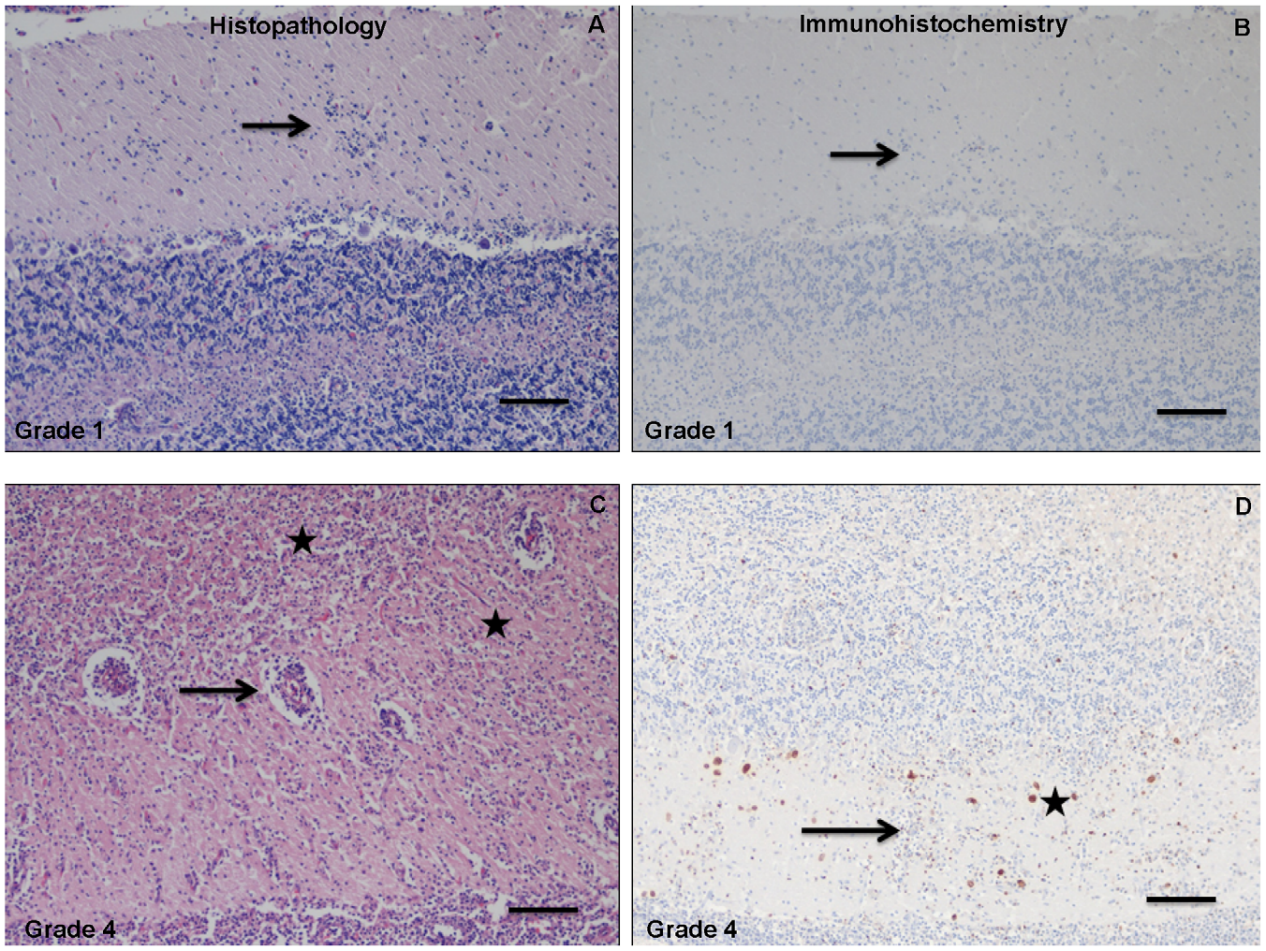
Histopathological grading and immunohistochemical analysis of protozoal disease associated with mild and severe encephalitis. Brain sections, stained with haematoxilin and eosin underwent histopathologic investigation (A,C) and immunohistochemistry (B,D) to detect the presence of *T. gondii* and *S. neurona*. Degree of protozoal encephalitis was scored (0: absent, 1–2: mild to moderate, 3–4: marked to severe) based on extent and severity of protozoal encephalitis and the presence and number of parasites. (A,B) show a harbor seal with mild protozoal encephalitis (grade 1), and (C,D) show a harbor seal with severe protozoal encephalitis (grade 4). (A) Mild encephalitis with scattered clusters of inflammatory cells within the molecular layer of the cerebellum. Arrow indicates inflammatory cells. (B) Immunohistochemical staining for *S. neurona* in the inflammatory infiltrate of the same animal. No discernible parasite-binding antigen is associated with the inflammation. Arrow indicates inflammatory cells. (C) Severe nonsuppurative and necrotizing meningoencephalitis of the cerebellum with effacement of the molecular and granular layers. Arrow details diffuse perivascular cuffing. Stars mark severely affected regions of the neuropil. (D) Immunohistochemical staining for *S. neurona* in the cerebellum of the same animal. Parasite-binding antigen is interspersed throughout the inflammatory and necrotic foci. Star marks dark brown deposits associated with parasite presence. Arrow details a focus of inflammation within the molecular layer of the cerebellum. Bar designates 10 µm for all images.

Diagnostic confirmation of *T. gondii* and/or *S. neurona* as the causal agent(s) of encephalitis and mortality was based on a combination of clinical signs and a positive molecular (PCR) result or, alternatively, histopathology, the presence of intralesional protozoa, and a positive PCR result. The immediate cause of death of animals with a positive PCR result but minimal to no apparent inflammation within examined sections of the brain was attributed to factors other than protozoal parasitism. For all PCR positive animals that had moderate to severe inflammation (Fig. 4), the degree of protozoal encephalitis and the presence of proliferating parasites was sufficient to explain the ante-mortem clinical signs and/or mortality of these animals [40].

**Figure 5:**
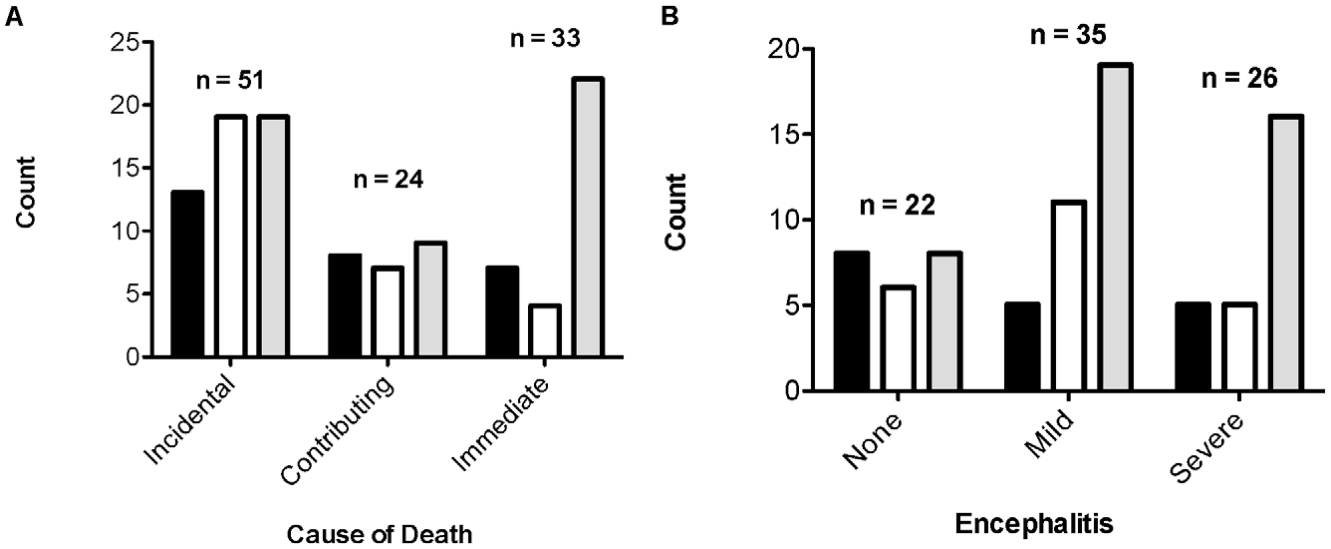
Pathological assessments demonstrate an association of polyparasitism with increased severity of infection. (A) Protozoal disease was identified as an incidental, contributing, or immediate cause of death for 108 stranded animals. The number of animals classified in each category is represented for single *S. neurona* infections (black), single *T. gondii* infections (white), and concomitant infections of *S. neurona* and *T. gondii* (grey). (B) Protozoal encephalitis, a manifestation of protozoal disease, was ranked as absent (score  = 0), mild (score  = 1–2) or severe (score  = 3–4) for 83 individuals. The number of animals classified in each category is represented as in (A). Sample sizes for each category are given. Graphs generated in Prism v5.0a.

One hundred and eight PCR-positive animals had brain sections that were of suitable integrity for histologic grading to determine whether protozoal infection was an immediate, contributing, or incidental cause of encephalitis and death. As a whole, protozoal disease was an immediate cause of death for 33/108 (31%) individuals. More specifically, protozoal parasitism was identified as an immediate cause of death for 7 out of 28 (25%) single *S. neurona* infections and 4 out of 30 (13%) single *T. gondii* infections. Twenty-two of 50 (44%) dual infections were reported to be an immediate cause of death (Fig. 5a). Accordingly, protozoal infection was significantly more likely to be diagnosed as an immediate cause of death in a dual versus a single infection (χ2 = 6.695, DF = 1, p = 0.0091). Histopathology and immunohistochemistry analyses were systematically carried out on submitted samples from 2006–2009. In the majority of dual infections during this time period, *S. neurona,* rather than *T. gondii,* was identified as the predominant parasite proliferating in the tissue sections examined (data not shown).

A subset of these individuals (n = 83) were assessed for degree of protozoal encephalitis, an inflammation of the brain associated with protozoal disease. Each case was ranked on a scale of 0–4, indicating absent, mild, moderate, marked, and severe encephalitis, respectively (Fig. 4). Overall, protozoal disease was associated with severe/marked encephalitis in 26/83 (31%) individuals. In accordance with higher rates of mortality, dual infections of *S. neurona* and *T. gondii* correlated with higher rates of severe/marked encephalitis (16 out of 43 cases, 37%) than the total of single *T. gondii* and *S. neurona* infections (5 each, or 10 out of 40 cases, 25%) (Fig. 5b). Statistically, the rates of severe or marked encephalitis did not differ significantly between single versus dual infections (χ2 = 0.9245, df = 1, p = 0.3363).

Geographic site of infection also correlated with severity of protozoal disease. When resident populations from the outer coast were compared with those from the inland waters, the outer coast marine mammals had significantly higher rates of protozoal disease diagnosed as an immediate cause of death (χ2 = 9.6246, df = 1, p = 0.002) and significantly higher rates of severe/marked encephalitis (χ2 = 4.0320, df = 1, p = 0.045), despite similar infection profiles across the two sites (Fig. 1b).

### Genetic structure of Toxoplasma gondii infection

**Figure 6:**
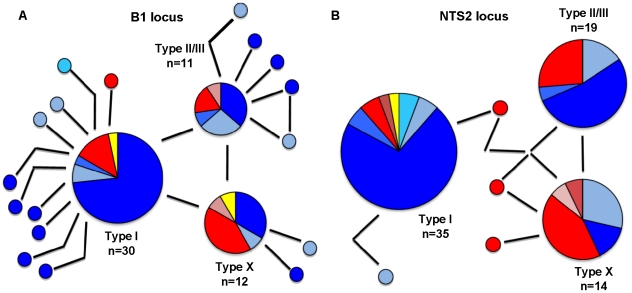
Type I alleles dominate *Toxoplasma gondii* genotypes infecting marine mammals of the Pacific Northwest. Network diagrams of genotyped *T. gondii* infections at the *B1* (n = 70) (A) and *NTS2* (n = 72) (B) loci. Chart size is directly proportional to the allele frequency. A single solid circle marks one individual. A single black bar indicates one nucleotide change; a kinked bar indicates that 2 nucleotide changes distinguish alleles. Colors and shadings are used to denote the host species from which a given allele was genotyped. Shades of blue indicate species from the suborder Pinnipedia: Harbor seal (royal blue), Guadalupe fur seal (pale blue), Steller sea lion (sky blue), and Elephant seal (teal). Shades of red mark species from the order Cetacea: Harbor porpoise (red), Killer whale (rose), and Pygmy sperm whale (maroon). Yellow denotes alleles derived from infections of Northern sea otters. Frameworks for diagrams were constructed in TCS v1.21.

DNA sequences for one to three *T. gondii* loci (*B1, NTS2, SAG1*) were obtained for 85 individuals. Thirty-seven (45%) possessed alleles consistent with a Type I or Type I-like genotype (Table S2), and Type I and Type-I like alleles dominated at the *B1* (42/74, 57%) and *NTS2* loci (41/78, 53%). (Fig. 6, Table S2). Figure 6 shows a network estimation diagram, generated in TCS v1.21, of the relationships between the alleles in the *T. gondii* population; the direct proportionality of chart size to allele frequency reveals the over-representation of Type I alleles at both the *B1* and *NTS2* loci. At the *B1* locus, 23% (17/74) of infections carried Type II/III or II/III-like alleles and 20% (15/74) were characterized by Type X or X-like alleles. Type II/III alleles represented 26% (20/78) of alleles at the *NTS2* locus, and Type X or X-like alleles represented 21% (16/78) (Table S2). Independent amplifications of multiple tissues per animal demonstrated definitively that 11 individuals (13%) were infected by multiple genotypes possessing distinct tissue tropisms. An additional 22 individuals (26%) were infected by multiple genotypes or by single genotypes harboring unique alleles; our typing analyses were not capable of readily distinguishing between these alternatives (Table S2).

*Toxoplasma gondii* genotype (as defined by the concatenated genotypes in Table S2) was not significantly associated with immediate cause of death or with development of marked/severe protozoal encephalitis (Fisher' Exact, p = 0.1953; p = 0.3644, respectively). Nor did mixed *T. gondii* infections differ significantly from single genotype infections in the rates of protozoal disease reported as an immediate cause of death (Fisher' Exact, p = 0.7055) or in the rates of severe/marked encephalitis (Fisher' Exact Test, p = 1) (Table S2). However, further sampling is required before definitive conclusions can be drawn from these analyses, due to the low samples sizes in this data set.

At both the *B1* and *NTS2* loci, pinnipeds were significantly more likely to be infected by genotypes carrying Type I lineage alleles than cetaceans, which were significantly more likely to be infected by genotypes carrying Type X alleles (*B1*: Fisher' Exact test, p = 0.01890; *NTS2*: Fisher' Exact test, p = 0.0001156). This distribution pattern is readily visualized in Figure 6, where allelic populations are presented in shades of blue to represent pinniped species and in shades of red to represent cetacean species. However, sample sizes were again insufficient to fully analyze this trend and to control for potentially confounding factors such as year of collection.

## Discussion

Polyparasitism commonly occurs in human and wildlife populations and has been found to influence the severity of disease [1], [2], [6], [18], [19]. The generalist parasite *T. gondii* infects essentially all warm-blooded vertebrates globally, and this high prevalence in nature suggests that it commonly co-occurs with other infectious agents. Parasite genotype has been identified as one intrinsic factor governing the course of *T. gondii* infection [7], [8], and this 6-year study shows polyparasitism to be another important contributor to the severity of protozoal disease. Here, we show an association of polyparasitism with symptomatic and fatal protozoal disease in natural populations of marine mammals of the Pacific Northwest, the site of one of the largest outbreaks of *T. gondii* in humans [31], [43].

Of 161 marine mammals investigated, 91% were found infected with tissue-encysting coccidian parasites. Between 2004 and 2009, over 6000 stranded marine mammals were documented in the Pacific Northwest. The majority of marine mammals in this study represent a subset of these strandings that were reported as suspect protozoal cases. Our sampled population is thus likely biased towards increased detection of protozoal infection. However, the 100% infection rate in the 10 healthy adult California sea lion males included in this study argues that such frequent infection with a diversity of protozoal parasites may extend to populations of apparently healthy marine mammals. This study finds significantly higher rates of infection than similar serological and histopathological population-level surveys conducted in California and the Pacific Northwest, where estimates of protozoal prevalence in marine mammals ranged from 7 to 62% [27], [28], [40], [44]–[47]. Ultimately, to determine the true population-level prevalence of protozoal infection in wild marine species of the Pacific Northwest, sampling additional healthy, asymptomatic individuals will be required. Importantly, the genetic tools developed herein provide a sensitive method for detecting both acute and low burden chronic infections to complement other less sensitive, serological approaches. They also provide an unbiased history of infections present in marine mammals, as the direct PCR-DNA sequencing technique is not subject to selection bottlenecks for only those strains that can be recovered by bioassay through mice or by in vitro propagation.

Molecular genotyping of *T. gondii* infections revealed an abundance of genotypes possessing Type I and Type I-like lineage alleles (45%) (Fig. 6), as well as multiple mixed genotype infections (13%) and those bearing atypical lineage alleles (26%) (Table S2). This is in striking contrast to the prevailing consensus that humans and domestic animals are most commonly singly infected with strains of clonal Types II or III in North America; the Type I lineage is considered relatively rare and has a highly virulent phenotype in laboratory mouse models [8], [48]. The prevalence of Type I alleles in the Pacific Northwest is consistent with the identification of a genotype bearing Type I lineage alleles associated with the 1995 human toxoplasmosis outbreak, and with recent findings of Type I-like genotypes circulating in avian wildlife in the region [49], [50]. Whether these are in fact clonal Type I strains will, however, require extensive PCR-DNA sequencing utilizing additional markers on parasite isolates recovered from marine mammals and other wildlife in the region.

Similar surveys have linked clonal Type II and the Type X clade of strains to marine mammal mortality events in California [17], yet these genotypes were not the dominant ones identified in the Pacific Northwest population (Oregon, Washington, and southern British Columbia). The distribution of genotypes identified in this study is thus unlikely to have resulted from sampling of predominantly dead, stranded individuals. Rather, the divergent populations of genotypes emerging in these two regions of the North American Pacific Coast suggest that spatially distinct transmission dynamics define the population structure of *T. gondii*. Interestingly, we found no significant relationship between parasite genotype and disease severity, as measured by association with mortality and protozoal encephalitis. This indicates that other factors, including polyparasitism, are important contributors to the severity of protozoal disease in the studied population.

In this study, concomitant infections with *T. gondii* and *S. neurona* were very common (42%) (Fig. 1), which is consistent with their having shared routes of transmission (water/food-borne) [51]. This high frequency indicates that prior infection with one does not result in immunological exclusion of the other, despite their relatedness. This finding coincides with previous studies that report frequent intraspecific multiple infections with 2 or more genotypes of *T. gondii* [52], [53] and with Thomas' [40] report of 30% dual infection of *T. gondii* and *S. neurona* in sea otters with protozoal encephalitis. It also suggests that the risk factors for *T. gondii* and *S. neurona* infection in marine mammals of the Pacific Northwest are not so divergent as to preclude concomitant infection, as reported by Johnson [45]. These high rates of infection and polyparasitism illustrate a serious public health issue given that coastal marine environments are major sources of food and water for humans, wildlife, and domestic animals [54]. Toxoplasmosis has already been recognized to be a prominent parasitic disease among indigenous Arctic populations due to their regular consumption of marine mammal meat and contaminated drinking water [54]. Marine mammals have previously demonstrated their value as sentinels for the detection of emerging zoonotic agents, most notably in the ongoing emergence of a highly virulent clade of *Cryptococcus gattii* in animals and humans of the Pacific Northwest [25], [55]. Here, the prevalence of concomitant infection in marine sentinel species indicates a potential role for polyparasitism in the emergence of protozoal disease in other host species, including humans. Accordingly, in regions of the world where *T. gondii* and human *Sarcocystis* species are coendemic (e.g. Southeast Asia), the prevalence of human polyparasitism merits investigation [56], [57].

In our data set, acute and chronic infections, as defined by pathological assessments of mortality and protozoal encephalitis, were attributed to both single and dual infections. Our finding of many chronic, asymptomatic *S. neurona* single infections argues that *S. neurona* is not intrinsically virulent to marine mammals, as reported in several studies [29], [40]. In fact, the majority (8/10) of the healthy California sea lions included in this study were singly infected with *S. neurona*. Of the most virulent protozoal disease cases in this report, the majority were linked to polyparasitism. Pathology grading found that single infections of *T. gondii* were least frequently associated with protozoal infection as an immediate cause of death, whereas *S. neurona* single infections were least frequently associated with severe protozoal encephalitis (Fig. 5).

These patterns in marine mammals coincide with those of human disease, where *T. gondii* and *Sarcocystis* species have been linked to both benign, latent infections and severe disease [7], [56], [58]. Conversion of a chronic, asymptomatic infection to acute disease (i.e. recrudescence) occurs commonly in *T. gondii*, as evidenced by fatal toxoplasmosis in HIV/AIDS and other immunosuppressed patients [22]. Likewise, immunosuppression associated with exhaustion, pregnancy, and stress is linked to severe protozoal encephalitis in *S. neurona*-infected horses [59], [60]. Accordingly, cases presenting with acute protozoal disease in our marine mammals likely reflect recrudescence of chronic infections induced by immunosuppression due to mating, pregnancy, and pupping. It is also conceivable that polyparasitism represents a greater challenge to the immune system than a single infection, and this may explain the higher rate of severe disease seen in polyparasitized marine mammals. Moreover, the effect of immunosuppression and the frequency of recrudescence may be amplified in marine mammals due to environmental pollutants (i.e. PCBs, DDT) that concentrate at high levels in the marine environment and compromise cellular and humoral immunity [23], [26], [61]–[66]. Spatial variation in these environmental factors may help to explain the striking differences in severity of protozoal disease between resident marine mammals of the outer coast and inland waters, despite these two populations having very similar infection profiles [63], [66].

Marine mammals are also infected with an increasing diversity of pathogenic agents [23], [26], [62], which is supported by our finding of DNA sequences consistent with the discovery of eight previously undescribed tissue-encysting coccidia for which host range and disease potential are unknown (Fig. 2). Further investigation of these unique coccidia and the pathology associated with their infection is warranted, particularly in light of the relationship of polyparasitism and severe disease found in this study. Broadly, this system powerfully demonstrates that disease is modulated at many levels, and the interaction of host microbial community, parasite and host genotypes, and environmental pollutants demands future investigation.

Finally, order of infection may be a significant factor determining the severity of concomitant infections. Experimental coinfections of *T. gondii* with the parasites *Schistosoma mansoni, Leishmania major,* and *Nippostrongylus brasiliensis* have found that disease severity is strongly linked with order of infection [21], [67]–[69]. The role order of infection plays cannot be directly tested by our study. However, work by Thomas [40] observed that *S. neurona* was the more aggressive parasite in *T. gondii*/*S. neurona* coinfections associated with severe protozoal disease. This coincides with analyses from this study that implicate *S. neurona* as the proliferating parasite in the majority of dual infections from 2006 to 2009. Accordingly, superinfection of a chronically *T. gondii-*infected animal with *S. neurona* may yield recrudescence of *T. gondii* and a more severe case of protozoal disease. This hypothesis could be tested by determining the relative levels of parasite-specific anti-IgM versus IgG levels among stranded marine mammals carrying dual infections. Moreover, our results demonstrate that the environmental load of *S. neurona* has changed dramatically over the brief time span of this study, with *S. neurona* emerging as an important pathogen infecting marine wildlife in the Pacific Northwest over just the last 8 years. If indeed order of infection is a key factor in concomitant infection, this fluctuation in parasite populations over time has important implications for disease severity across host environments.

The mechanisms that generate severe infection, rather than chronicity, are not clearly defined for humans infected with tissue-encysting coccidia. In the case of *T. gondii,* disease varies widely, and no large-scale epidemiological study, with multilocus genotyping and healthy controls, has been conducted to infer associations between strain type and human disease [7], [11], [14], [70]. In fact, this study did not find an association of strain type with disease severity, though low sampling indicates that additional research into whether parasite genotype is associated with disease in marine mammals is still warranted. Rather, we identified polyparasitism as a new variable associated with disease severity in protozoal infection. Species of *Sarcocystis* are far less studied, though they are as widespread as *T. gondii* and infect many of the same mammalian hosts, including humans [56], [71]. As mirrored in our population of marine mammals, *Sarcocystis* leads to benign, chronic infections in humans. For example, *Sarcocystis* seropositivity in Malaysians is reported to be >20%, with the vast majority of positive cases being incidental diagnoses [56], [57], [72]. Exceptions do occur, however, such as the acutely virulent *Sarcocystis* outbreak in US military personnel stationed in Southeast Asia [58]. Our findings in marine mammals of symptomatic and fatal protozoal disease associated with concomitant infection of *Toxoplasma* and *Sarcocystis* present polyparasitism as a significant cofactor explaining this wide variation in disease severity. The role of polyparasitism in human infections has not been assessed and could serve as a relevant risk factor in regions of Southeast Asia where coccidian species are coendemic and overlap with environmental factors (i.e. malnutrition, poor sanitation), other NTDs, and the ‘big three’.

The emergence of *T. gondii* and *S. neurona,* and their association with protozoal disease in sentinel marine mammal species, points to pathogen pollution of waterways as a serious public health and conservation threat. Seasonal spikes in freshwater runoff have resulted in waterborne transmission of *T. gondii* and *S. neurona* to both humans [73], [74] and sea otters [46], [74]. Reduction of run-off, erosion, and urban pollution in coastal areas would therefore be an appropriate preventative measure. The previous occurrence in the Pacific Northwest of one of the largest outbreaks of *T. gondii* in humans, due to fecal contamination of a drinking water reservoir, necessitates vigilant surveillance and thorough treatment of public water sources [31], [43], [73]. Moreover, we demonstrate the potential for rapid increases in protozoal prevalence in marine sentinels, suggesting surges in the risk of human exposure and variation in the potential for polyparasitism. As *T. gondii* and other protozoal pathogens continue to emerge, this study demonstrates that polyparasitism serves as a critical factor contributing to the severity of protozoal disease in marine wildlife.

## Supporting Information

Table S1 Accession numbers for Genbank reference sequences of the ITS1 locus used in phylogenetic reconstruction.
(PDF)

Table S2 Concatenated genotypes, based upon sequencing at three loci, and pathology grading for *Toxoplasma gondii* infections.
(PDF)
