# Supplementary material for: Polyparasitism Is Associated with Increased Disease Severity in Toxoplasma gondii-Infected Marine Sentinel Species
Source: PLoS Negl Trop Dis. 2011 May 24;5(5):e1142. doi: 10.1371/journal.pntd.0001142 (PMC3101184; doi:10.1371/journal.pntd.0001142)
Supplement: Table S1 — Accession numbers for Genbank reference sequences of the ITS1 locus used in phylogenetic reconstruction. (PDF) [file pntd.0001142.s001.pdf]

Table S1: Accession numbers for Genbank reference sequences of the ITS1 locus used in phylogenetic reconstruction

Species	Accession Number	Reference
Besnoitia besnoiti	FN257464	Kiehl et al. (2010)
Hammondia hammondi	AF096499	Liddel et al. (1998)
Neospora caninum	AF038861	Marsh et al. (1998)
Sarcocystis canis	DQ176645	Dubey et al. (2006)
Sarcocystis cruzi	EF622176	Rosenthal et al. (2008)
Sarcocystis neurona	AY082648	Cheadler et al. (2002)
Toxoplasma gondii	AF252408	Miller et al. (2001)
Coccidia C	GU936628	Present manuscript
HS29	HQ184186	Present manuscript
GFS2	HQ184187	Present manuscript
GFS3	HQ184188	Present manuscript
SW1	HQ184185	Present manuscript
