# Supplementary material for: Polyparasitism Is Associated with Increased Disease Severity in Toxoplasma gondii-Infected Marine Sentinel Species
Source: PLoS Negl Trop Dis. 2011 May 24;5(5):e1142. doi: 10.1371/journal.pntd.0001142 (PMC3101184; doi:10.1371/journal.pntd.0001142)
Supplement: Table S2 — Concatenated genotypes, based upon sequencing at three loci, and pathology grading for Toxoplasma gondii infections. (PDF) [file pntd.0001142.s002.pdf]

Table S2: Concatenated genotypes, based upon sequencing at three loci, and pathology grading for *Toxoplasma gondii* infections

Identification #	*T. gondii* genotype	*NTS2* allele	*B1* allele	*SAG1* allele	Cause of Death	Encephalitis Score
CSL3	I	I			Incidental	
HP12	I	I	I		Incidental	
HP14	I	I	I		Incidental	0
HS8	I		I		Incidental	
HS9	I	I			Contributing	
HS11	I	I			Immediate	2
HS16	I	I			Incidental	2
HS17	I		I		Immediate	4
HS18	I	I	I		Immediate	3
HS19	I	I	I		Contributing	3
HS20	I		I		Incidental	0
HS23	I	I			Incidental	
HS28	I	I			Incidental	1
HS37	I	I			Immediate	3
HS38	I	I			Immediate	3
HS39	I	I			Immediate	3
SO7	I	I	I	I		
CSL12	I	I			Incidental	2
HS46	I		I	I	Contributing	1
HS50	I	I	I		Immediate	3
SSL4	I	I			Contributing	3
HS56	I		I		Immediate	3
HS61	I		I		Contributing	2
PSW2	I	I	I		Incidental	
HS67	I	I	I	I	Contributing	2
HS73	I	I			Contributing	
SSL6	I	I	I	I	Incidental	1
HS74	I		I		Contributing	
HS79	I		I		Incidental	0
HP9	I		U_I		Incidental	
HS13	I	I	U_I		Immediate	2
HS14	I	I	U_I		Immediate	3
GFS9	I	I	U_I		Incidental	
HS57	I		U_I		Immediate	3

HS62	I		U_{I}		Incidental	1
HS72	I	I	U_{I}	U_{I}	Immediate	4
ES1	I		U_{I}		Incidental	
HP10	II	II/III	II/III	II/III	Immediate	
HP11	II	II/III	II/III	II/III	Immediate	2
HP18	II	II/III			Immediate	2
HS26	II	II/III			Incidental	0
GFS3	II	II/III			Contributing	
GFS10	II		II/III		Incidental	1
HS81	II	II/III	II/III		Immediate	3
SSL8	II	II/III	II/III		Incidental	0
HP27	X	X	X		Contributing	
HP28	X	X			Incidental	
HS25	X		X		Contributing	2
GFS5	X	X	X			
GFS7	X	X				
HS59	X		X		Contributing	1
HP37	X	X	X			0
HS12	Mixed Infection: I, II	I	I / II/III	I / II/III	Incidental	0
HS49	Mixed infection: I, II	I	I / II/III	I	Immediate	4
GFS11	Mixed infection: I, II	I	U_{I} / $U_{II/III}$		Contributing	2
GFS13	Mixed Infection: I, II	U_{I}	I / $U_{II/III}$	I / II/III	Incidental	1
SSL7	Mixed infection: I, II	I / II/III	I		Incidental	0
HS75	Mixed infection: I, II	I / II/III			Immediate	3
HS77	Mixed infection: I, II	I / II/III	I / $U_{II/III}$	I	Incidental	
HP33	Mixed Infection: I, X	X	I / X		Incidental	0
HS7	Mixed Infection: I, X	I	I / X		Incidental	2
PSW1	Mixed Infection: I, X	I / X	X		Incidental	0
HS83	Mixed Infection: II, X	II/III / X	II/III		Immediate	3
HS3	Atypical	II/III	I	II/III	Contributing	2
HS6	Atypical	I	I / $U_{II/III}$		Incidental	0
HS10	Atypical	I / II/III	U_{I}		Contributing	1
HS21	Atypical	II/III	I			
HS30	Atypical	I	$U_{II/III}$	I	Incidental	
GFS4	Atypical	I / II/III	II/III		Immediate	2
HS60	Atypical	I / II/III	I	I	Immediate	2
HS66	Atypical	II/III	I		Contributing	

HS69	Atypical	II/III	I	I		
KW1	Atypical	X	II/III X			
HP21	Atypical	U_X		II/III X	Immediate	3
GFS1	Atypical	II/III	U_X			
GFS6	Atypical	X	II/III			
HP39	Atypical	X	X	II/III	Incidental	0
HP40	Atypical	II/III X	X	II/III	Incidental	0
HP5	Atypical	U_X	I	X	Incidental	0
GFS2	Atypical	X	I			
HS58	Atypical	I	U_X	I	Immediate	4
HS76	Atypical	X		I	Immediate	3
HS4	Atypical	I	II/III X		Incidental	1
HP4	Atypical	**U**			Contributing	1
SO2	Atypical		X	**U**	Incidental	

[a] Identification numbers also indicate species identity for each individual: CSL = California sea lion, HP = harbor porpoise, HS = harbor seal, SO = Northern sea otter, SSL = Steller sea lion, PSW = Pygmy sperm whale, GFS = Guadalupe fur seal, ES = Elephant seal, KW = Killer whale.
[b] Type I and Type I-like alleles are shaded in black, Type II/III and Type II/III-like alleles in dark grey, Type X and Type X-like alleles in pale grey, and unique alleles in white.
[c] Sequences were classified as archetypal I, II/III or X or as Type I-like, II/III-like or X-like alleles, (represented by $U_{I,}$ $U_{II/III,}$ or U_X, respectively) when polymorphisms were present in addition to allele-defining archetypal polymorphisms. A sequence was identified as a unique allele (U) when it was sufficiently divergent from archetypal alleles.
[d] Mixed infections are infections for which multiple genotypes with distinct tissue tropisms were amplified from a single host. Atypical infections are infections of genotypes with unique alleles and/or unique combinations of alleles or of multiple genotypes.
[e] “Cause of Death” and “Encephalitis Score” columns provide pathology grading, when available. “Cause of Death” indicates whether protozoal disease was an immediate, contributing, or incidental cause of death. “Encephalitis Score” ranges from absent (0) to severe (4) and indicates the severity of protozoal encephalitis.
